# Traditional Chinese medicine in the management of diabetic foot ulcers: an overview of meta-analyses

**DOI:** 10.3389/fmed.2025.1651966

**Published:** 2025-09-17

**Authors:** Xiaocui Xiong, Xiaoman Liu, Siyuan Wan

**Affiliations:** Department of Rehabilitation and Traditional Chinese Medicine, Hospital of Wuhan University, Wuhan, China

**Keywords:** traditional Chinese medicine, herbal therapy, diabetic foot ulcer, umbrella review, Chinese medicine

## Abstract

**Background:**

The global rise in diabetic foot ulcer (DFU) complications necessitates more effective therapeutic strategies. Traditional Chinese Medicine (TCM) has been increasingly explored as a potential adjunctive therapy for DFU management. This umbrella review synthesizes evidence from meta-analyses to evaluate the effectiveness and safety of TCM interventions.

**Methods:**

A systematic search of PubMed, Scopus, and Web of Science was conducted through May 2025. Eligible meta-analyses were selected based on predefined criteria, and methodological quality was appraised using the AMSTAR 2 tool.

**Results:**

Eleven meta-analyses, encompassing 44 datasets and reporting pooled effect sizes for seven clinical outcomes, were included. The TCM interventions analyzed were both topical and oral. The findings revealed that TCM significantly improved DFU-related outcomes, including ulcer size reduction, shortened healing time (by up to 5.7 days in some trials), increased cure rates (up to a 43% relative improvement), and enhanced overall treatment effectiveness. Notably, longer intervention durations and larger sample sizes were associated with stronger positive effects.

**Conclusion:**

This review underscores the therapeutic potential of TCM, particularly topical formulations, in enhancing clinical outcomes and accelerating recovery in patients with diabetic foot ulcers.

## 1 Introduction

Diabetes mellitus (DM) is a chronic metabolic disorder characterized by hyperglycemia resulting from various pathogenic factors (1). According to the American Diabetes Association, DM is classified into four types: type 1, type 2, gestational diabetes, and diabetes due to other causes (2). The global prevalence of DM has risen markedly in recent decades. In 2017, approximately 451 million people were estimated to have DM—predominantly type 2—with projections reaching 693 million by 2045 (3, 4). The primary therapeutic goal in type 2 diabetes is to prevent or delay complications and maintain quality of life (5).

One of the most serious complications is diabetic foot ulcer (DFU), a chronic, non-healing wound commonly occurring on the feet or lower legs of individuals with diabetes. DFU results from multiple contributing factors, including peripheral neuropathy, peripheral arterial disease, trauma, foot deformities, poor glycemic control, impaired immune function, and delayed wound healing (6–8). Neuropathy, characterized by sensory loss and reduced proprioception, increases susceptibility to trauma and pressure, heightening ulcer risk. DFU represents a significant global health concern, with an estimated annual incidence of 9.1 to 26.1 million cases and a lifetime risk of 15–25% among individuals with diabetes (9).

DFU complications—including infection, cellulitis, osteomyelitis, gangrene, amputation, and increased mortality—often stem from impaired circulation and immune dysfunction (6, 10). Traditional Chinese Medicine (TCM), a holistic system with over 2,000 years of history, offers an integrative approach to managing such conditions. TCM includes herbal medicine, acupuncture, dietary therapy, qigong, and massage (tui na), and is founded on the concept of restoring balance in the body’s vital energy (qi) via meridians (11). TCM has been increasingly applied in the management of DFU through herbal remedies, acupuncture, and lifestyle interventions, aiming to improve circulation, reduce inflammation, and enhance wound healing (12).

The emergence of bioinformatics has enhanced the scientific understanding of Traditional Chinese Medicine (TCM), providing insight into its underlying mechanisms. Recent research has demonstrated significant progress in applying TCM to chronic non-communicable diseases (13). Notably, certain herbal formulations—such as those containing Huang Qi—have been shown to improve insulin sensitivity, regulate blood glucose, and reduce the risk of diabetes-related complications, including neuropathy and cardiovascular disease (14, 15). Specifically, evidence suggests that oral or injectable herbal therapies, when used as adjuncts to conventional treatments, can enhance healing in patients with DFU (16, 17).

Several meta-analyses have assessed the effects of various TCM interventions on DFU-related outcomes, including ulcer area, cure rate, effectiveness rate, adverse events, healing time, hospitalization duration, and amputation rate (16–25). However, these studies have reported varying results and effect sizes. Prior reviews typically focused on single TCM formulas or outcomes and rarely contrasted delivery forms (topical vs. oral), providing limited guidance across modalities. Recent international reports also highlight growing interest in TCM-based adjuncts for DFU management beyond China underscoring the need for a comprehensive umbrella review of existing meta-analyses (26–29). To fill this gap, our umbrella review collates and compares meta-analyses of randomized controlled trials (RCTs) across multiple TCM interventions, synthesizes intervention-specific effects by outcome, and interprets findings in light of methodological quality (AMSTAR 2). This approach offers a comparative, quality-aware map of effectiveness and safety to inform practice and future trials.

## 2 Methods

The study followed the Preferred Reporting Items for Systematic Reviews and Meta-analyses (PRISMA) reporting guideline (30).

### 2.1 Search strategy

We searched PubMed, Scopus, and Web of Science (WoS) from inception to May 2025, with supplementary checks in Google Scholar. Search strings combined DFU terms with TCM terms and a meta-analysis filter (full strategies in Supplementary file). Field limits were: PubMed Title/Abstract, Scopus TITLE-ABS-KEY, and WoS Topic, which searches Title, Abstract, Author Keywords, and Keywords Plus. No date limits were applied. Language was restricted to English at the umbrella-review level. We screened reference lists of eligible papers and related reviews for additional studies.

### 2.2 Inclusion and exclusion criteria

The PICO criteria for the present umbrella meta-analysis were structured as follows: Population/Patients (P), eligible individuals were adults aged 18 years or older DFU; Intervention (I) focused on the administration of TCM including orally and topically; Comparison (C) control group or placebo; Outcome (O) DFU related outcomes including ulcer area, cure rate, effectiveness rete, healing time, adverse events, hospitalization duration and Amputation rate. Only meta-analysis studies published in English that explored the impact of TCM on DFU outcomes and had reported effect sizes (ES) along with their corresponding confidence intervals (CI) were considered for inclusion. No restriction was placed on the year of publication. Studies published in languages other than English were excluded, as our team was unable to ensure accurate translation and quality assessment across multiple languages. Original studies, editorials, letters to the editor, and observational studies, were excluded from consideration.

### 2.3 Study selection and data extraction

Studies were independently screened and Two reviewers (XL, SW) extracted the data based on pre-established criteria. This included author’s name, year of publication, sample size, study location, study number, TCM supplementation duration, intervention type, consumption type, mean age, Heterogeneity, ES and CI for DFU related parameters. Any discrepancies were resolved with discussion with third reviewer (XX).

### 2.4 Methodological quality assessment and data extraction

Two independent reviewers (XX, XL) assessed the methodological quality of the included articles using the Assessing the Methodological Quality of Systematic Reviews 2 (AMSTAR2) questionnaire (31). The AMSTAR2 questionnaire comprises 16 items that are answered with “Yes,” “Partial Yes,” “No,” or “Not a Meta-analysis.” The AMSTAR2 checklist is divided into four categories: “Critically low quality,” “Low quality,” “Moderate quality,” and “High quality.” A score of 7 or higher indicated that a meta-analysis was of high quality.

## 3 Result

### 3.1 Literature review

**Figure 1:**
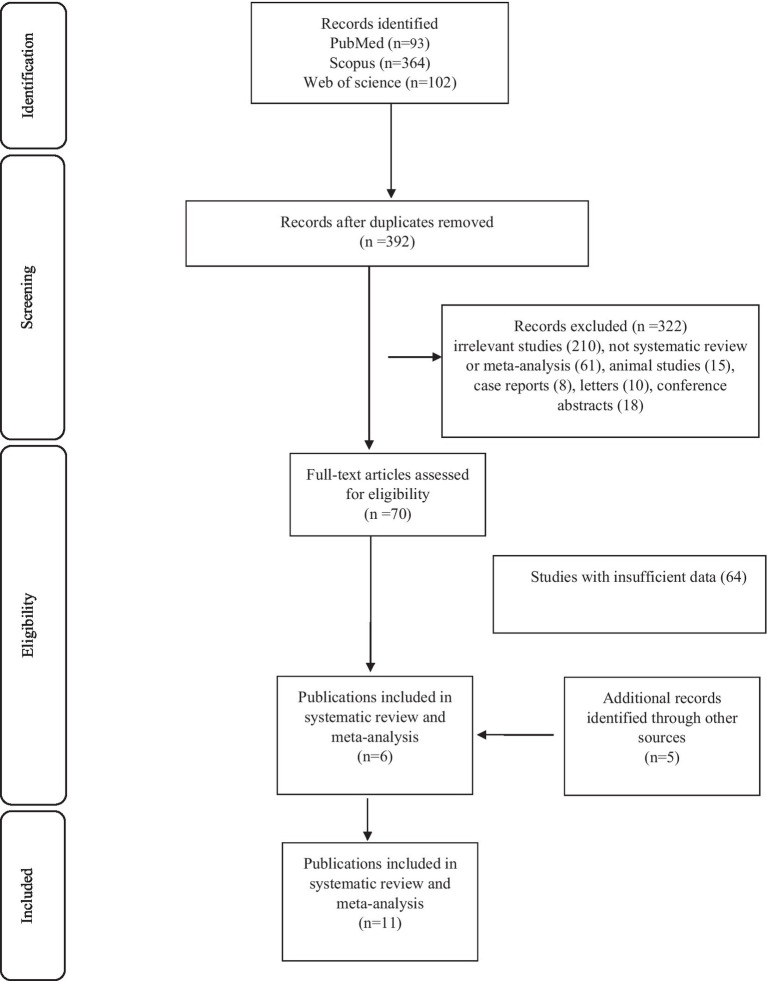
Flow diagram of study selection.

First, we retrieved 559 articles by searching databases. Second, 392 studies relevant to the effect of TCM on DFU outcome remained after deduplication. Then, after evaluating the titles and abstracts, 322 articles were excluded. Next, 64 studies were also excluded after the full-text screening. In the next step, 5 studies from the rest of the sources that met the inclusion criteria were entered. Finally, a total of 11 studies with 44 data-sets were regarded as eligible for the umbrella review (Figure 1).

### 3.2 Characteristics of the included meta-analyses

The characteristics of 11 meta-analyses with 44 data sets are presented in Table 1. All studies have been conducted in China. All included meta-analyses synthesized RCTs; no eligible meta-analysis of purely observational primary studies was identified published from 2010 to 2024. Eight studies, including 15 data sets, were conducted to assess the effects of TCM on effectiveness rates (16, 18, 19, 22, 23, 25, 32, 33). Five studies, including eight data sets, have investigated the impact of TCM on the cure rate (19, 20, 23–25). A total of eight studies, including nine data sets, have examined changes in healing time as a dependent variable in response to the TCM (19, 20, 22–25, 32, 33). Five studies, including eight data sets, have been conducted to assess the effects of TCM on ulcer areas (17, 18, 20, 32, 33). A total of two studies, including two data sets, have examined changes in hospitalization duration as a dependent variable in response to the TCM (20, 22), one study has investigated the impact of TCM on Amputation rate (25), and finally one study including one data set has investigated the effect of TCM on adverse events (18). The sample size of the included data sets ranged from 86 to 1958 people. Also, the age range of the data sets was from 55 to 65 years, and the average duration of interventions was from 1.7 weeks to 27 weeks. Among the clinical trial studies included in the mentioned meta-analyses, in three articles, the interventions were oral (16–18), one study was both oral and topical (25), and the rest were topical (19, 20, 22–24).

**Table 1:** Characterization of included studies.

First author	year	Study number	Intervention type	Consumption type	No. intervention or exposure/control group	Outcome	Duration (week)	Effect size	Direction of effect	Age (mean)	Heterogeneity
Jian et al. (33)	2024	7	Chinese herbal medicine	Oral and topically	236/230	Ulcer area	NR	MD	Positive	NR	I_2 =_ 59%
		4			118/118	Healing time		MD	Positive		I_2 =_ 79%
		14			465/460	Effective rate		RR	Positive		I_2 =_ 36%
Ni et al. (32)	2024	2	Danggui Sini decoction	Oral	107/107	Ulcer area	NR	MD	Positive	60	I_2 =_ 68%
		2			78/78	Healing time		MD	Positive		I_2 =_ 75%
		5			204/200	Effective rate		RR	Positive		I_2 =_ 0%
Yang et al. (19)	2023	2	Astrogalin	Topically	118/125	Cure rate	3	RR	Positive	60	I_2 =_ 0%
		12	MEBO dressing		388/382	Cure rate	3.5	RR	Positive	65	I_2 =_ 66%
		9	Compound Cortex Phellodendri		1166/792	Cure rate	4.7	RR	No effect	55	I_2 =_ 34%
		6	Shengji Yuhong Ointment		242/238	Cure rate	4.5	RR	Positive	58	I_2 =_ 0%
		7	MEBO dressing		244/247	Healing time	4.3	SMD	Positive	58	I_2 =_ 95%
		2	Shengji Yuhong Ointment		58/58	Healing time	4	SMD	Positive	60	I_2 =_ 84%
		2	Astrogalin		118/125	Effective rate	3	RR	Positive	60	I_2 =_ 94%
		6	MEBO dressing		225/228	Effective rate	4	RR	Positive	55	I_2 =_ 88%
		7	Compound Cortex Chellodendri		1005/632	Effective rate	4.5	RR	No effect	55	I_2 =_ 72%
		6	Shengji Yuhong Ointment		242/238	Effective rate	4.5	RR	Positive	50	I_2 =_ 26%
Zhong et al. (18)	2023	2	Buyang Huanwu decoction combined with Western medicine	Oral	92/92	Ulcer area	NR	MD	No effect	NR	I_2 =_ 96%
		2	Buyang Huanwu decoction combined with Western medicine		68/56	Adverse events	6.5	RR	Positive	NR	I_2 =_ 0%
		17	Buyang Huanwu decoction combined with Western medicine		568/560	Effective rate	4.5	OR	Positive	55	I_2 =_ 0%
Feng et al. (20)	2022	3	Resina Draconis	Topically	134/127	Ulcer area	4	RR	Positive	55	I_2 =_ 0%
		3	Resina Draconis		89/82	Ulcer area	4.5	RR	Positive	58	I_2 =_ 0%
		6	Resina Draconis		259/240	Cure rate	4	RR	Positive	60	I_2 =_ 0%
		8	Resina Draconis		315/295	Healing time	4.5	MD	No effect	55	I_2 =_ 81%
		2	Resina Draconis		109/104	Hospitalization duration	3.75	MD	Positive	60	I_2 =_ 0%
Wan et al. (22)	2021	9	KangFuXin Liquid	Topically	350/346	Healing time	NR	SMD	Positive	55	I_2 =_ 93.4%
		11	KangFuXin Liquid		440/436	Effective rate	NR	OR	Positive	65	I_2 =_ 0%
		5	KangFuXin Liquid		204/204	Hospitalization stays	NR	SMD	Positive	65	I_2 =_ 97%
Qu et al. (23)	2019	5	KangFuXin Liquid	Topically	248/254	Cure rate	4	RR	Positive	NR	I_2 =_ 65%
		3	KangFuXin Liquid		151/150	Healing time	3	MD	Positive	NR	I_2 =_ 0%
		5	KangFuXin Liquid		239/244	Effective rate	3	RR	Positive	NR	I_2 =_ 32%
Tan et al. (16)	2018	6	Danhong injection	Injection	183/175	Effective rate	4	RR	Positive	58	I_2 =_ 0%
		3	Erigeron Breviscapus extract injection		117/115	Effective rate	2	RR	Positive	65	I_2 =_ 0%
		3	Compound *Salvia Miltiorrhiza* injection		148/148	Effective rate	4	RR	Positive	50	I_2 =_ 30%
		3	*Ginkgo biloba* extract injection		161/161	Effective rate	4	RR	No effect	60	I_2 =_ 0%
		2	Panax notoginsenosides injection		50/50	Effective rate	1.7	RR	Positive	59	I_2 =_ 0%
Chen et al (25)	2017	14	different traditional Chinese medication	Oral and topically	662/518	Cure rate	14.8	OR	Positive	60	I_2 =_ 0%
		5	different traditional chinese medication		182/182	Healing time	22.8	SMD	Positive	60	I_2 =_ 13%
		15	different traditional chinese medication		683/508	Effective rate	12.2	OR	Positive	60	I_2 =_ 0%
		4	different traditional chinese medication		152/147	Amputation rate	27	OR	Positive	60	I_2 =_ 7%
Ma et al. (24)	2018	4	Jinhuang powder	Topically	198 (all participations)	Cure rate	NR	RR	Positive	60	I_2 =_ 0%
		3	Jinhuang powder		NR	Healing time	NR	SMD	Positive	58	I_2 =_ 94.5%
Chen et al (17)	2010	3	Chinese herbal medicine	Oral	113/111	Ulcer area	4.5	RR	Negative	NR	I_2 =_ 76%
		4	Chinese herbal medicine		141/145	Ulcer area	5	RR	Negative	NR	I_2 =_ 0%
		6	Chinese herbal medicine		221/218	Ulcer area	7	RR	Negative	NR	I_2 =_ 0%

NR, Not reported; RR, Risk ratio; OR, odds ratio; SMD, standard mean difference; MD, mean difference; I^2^, heterogeneity statistic; Direction of Effect: Positive effect, significant beneficial effect in favor of TCM; No effect, non-significant result; Negative effect, significant reduction in outcome benefit.

### 3.3 Methodological quality assessment

Table 2 presents the findings of the quality assessment of meta-analyses according to the AMSTAR2 questionnaire. Among them, four studies received a high-quality score (16, 20, 24, 32), three received a moderate score (19, 21, 25), and five received a low score (17, 18, 22, 23, 33). Two studies in the search section used one researcher (19, 25), which may cause bias. Also, two studies have not examined the quality of the included studies and their risk of bias in their discussion (18, 22). Moreover, two studies did not assess publication bias or discuss its results (17, 23). None of the studies mentioned the funding of the included studies. On the other hand, five studies have mentioned the data related to clinical trials entered (dosage, duration of intervention, sample size, etc.) carefully and in detail (16, 17, 20, 22, 25). Also, four studies have searched entirely and accurately with reference checks (16, 20, 24, 25).

**Table 2:** Results of assess the methodological quality of meta-analysis.

First author	Q1	Q2	Q3	Q4	Q5	Q6	Q7	Q8	Q9	Q10	Q11	Q12	Q13	Q14	Q15	Q16	Overall
Jian et al. (33)	Yes	Yes	Yes	Partial Yes	Yes	Yes	Yes	Yes	Yes	No	Yes	Yes	No	No	Yes	Yes	low quality
Ni et al. (32)	Yes	Yes	Yes	Yes	Yes	Yes	Yes	Partial Yes	Yes	No	Yes	Yes	Yes	Yes	Yes	Yes	high quality
Zhong et al. (18)	Yes	Yes	Yes	Partial Yes	Yes	Yes	Yes	Partial Yes	Partial Yes	No	Yes	Yes	No	Yes	Yes	Yes	low quality
Yang et al. (19)	Yes	Yes	Yes	Partial Yes	No	Yes	Yes	Partial Yes	Yes	No	Yes	Yes	Yes	No	Yes	Yes	moderate quality
Feng et al. (20)	Yes	Yes	Yes	Yes	Yes	Yes	Yes	Yes	Yes	No	Yes	Yes	Yes	Yes	Yes	Yes	high quality
Wan et al. (22)	Yes	Yes	Yes	Partial Yes	Yes	Yes	Yes	Yes	Yes	No	Yes	Yes	No	No	Yes	Yes	low quality
Qu et al. (23)	Yes	Yes	Yes	Partial Yes	Yes	Yes	Yes	Partial Yes	Yes	No	Yes	Yes	Yes	Yes	No	Yes	low quality
Ma et al. (24)	Yes	Yes	Yes	Yes	Yes	Yes	Yes	Partial Yes	Yes	No	Yes	Yes	Yes	Yes	Yes	Yes	high quality
Tan et al. (16)	Yes	Yes	Yes	Yes	Yes	Yes	Yes	Yes	Yes	No	Yes	Yes	Yes	Yes	Yes	Yes	high quality
Chen et al. (25)	Yes	Yes	Yes	Yes	No	Yes	Yes	Yes	Yes	No	Yes	Yes	Yes	Yes	Yes	No	moderate quality
Chen et al. (17)	Yes	Yes	Yes	Partial Yes	Yes	Yes	Yes	Yes	Yes	No	Yes	Yes	Yes	Yes	No	Yes	low quality

Responses: Yes, Partial Yes, No, or Not applicable. Overall rating categories: high, moderate, low. Q: question; Q1- Did the research questions and inclusion criteria for the review include the components of PICO?; Q2- Did the report of the review contain an explicit statement that the review methods were established prior to the conduct of the review, and did the report justify any significant deviations from the protocol?; Q3- Did the review authors explain their selection of the study designs for inclusion in the review?; Q4- Did the review authors use a comprehensive literature search strategy?; Q5- Did the review authors perform study selection in duplicate?; Q6- Did the review authors perform data extraction in duplicate?; Q7- Did the review authors provide a list of excluded studies and justify the exclusions?; Q8- Did the review authors describe the included studies in adequate detail?; Q9- Did the review authors use a satisfactory technique for assessing the risk of bias (RoB) in individual studies that were included in the review?; Q10- Did the review authors report on the sources of funding for the studies included in the review?; Q11- If meta-analysis was performed, did the review authors use appropriate methods for the statistical combination of results?; Q12- If a meta-analysis was performed, did the review authors assess the potential impact of RoB in individual studies on the results of the meta-analysis or other evidence synthesis?; Q13- Did the review authors account for RoB in individual studies when interpreting/ discussing the review results?; Q14- Did the review authors provide a satisfactory explanation for and discussion of any heterogeneity observed in the review results?; Q15- If they performed quantitative synthesis, did the review authors conduct an adequate investigation of publication bias (small-study bias) and discuss its likely impact on the review results?; Q16- Did the review authors report any potential sources of conflict of interest, including any funding they received for conducting the review?

### 3.4 The effect of TCM on the effectiveness rate

Eight studies comprising 15 datasets examined the impact of various TCM interventions on effectiveness rates. Fourteen datasets reported a significant increase in effectiveness, while one—using *Astragalus membranaceus (Fisch.) Bunge (verified via The World Flora Online)*—showed no significant effect (Risk Ratio (RR): 1.70; 95% CI: 0.86–3.36; I^2^: 94%) (19). Significant heterogeneity was observed in three datasets from Yan-Wu Yang’s study (19) but not in the others. Da-Yuan Zhong et al. (18) found that *Buyang Huanwu* decoction, primarily based on *Astragalus membranaceus*, combined with Western medicine, yielded the highest effect size (OR: 6.12; 95% CI: 4.23–8.86; I^2^: 0%). Similarly, Xiaoping Wan et al. (22), reported a marked improvement with *Periploca sepium Bunge liquid* (RR: 5.38; 95% CI: 3.52–8.24; I^2^: 0%). In contrast, Lizi Tan et al. (16), observed the smallest increase using *Ginkgo biloba L*. extract injection (RR: 1.17; 95% CI: 1.08–1.27; I^2^: 0%).

### 3.5 The effect of TCM on cure rate

Five articles encompassing eight datasets assessed the impact of TCM on cure rate, all demonstrating a significant improvement. The greatest effect was observed in Yan-Wu Yang et al. (19), who used *Astragalus membranaceus* (Fisch.) Bunge (verified via The World Flora Online) as the intervention (RR: 2.37; 95% CI: 1.85–3.04; I^2^: 0%). In contrast, the smallest effect was reported by Li Ma et al. (24), using Jinhuang powder containing *Curcuma longa* L. (RR: 1.19; 95% CI: 1.06–1.33; I^2^: 0%). A large-scale study by Yan-Wu Yang et al. [21], involving 1,958 participants and using *Phellodendron amurense* Rupr, also showed a substantial increase in cure rate (RR: 2.01; 95% CI: 1.61–2.50; I^2^: 34%). Similarly, Chen Shuo et al.’s data set (25), with an average intervention duration of 14.8 weeks, reported a notable improvement (RR: 2.12; 95% CI: 1.63–2.77; I^2^: 0%). Significant heterogeneity was present in two datasets (19, 23), while the others showed no heterogeneity.

### 3.6 The effect of TCM on healing time

Eight studies comprising nine datasets evaluated the impact of various TCM interventions on healing time, all reporting significant reductions. The greatest reduction was observed in Ke Shen Qu (23), using *Periploca sepium* Bunge (verified via The World Flora Online; MD: –5.73; 95% CI: −6.95 to −4.52; I^2^: 0%), while the smallest was reported in Chen Shuo (standardized mean difference (SMD): –0.64; 95% CI: −0.89 to −0.40; I^2^: 13%), with an average intervention duration of 22.8 weeks—the longest among the studies. Both Xiaoping Wan and Ke Shen Qu employed *Periploca sepium* Bunge liquid (22, 23). The average intervention duration of the Chen Shuo dataset is 22.8 weeks, which is more than that of others (25). Significant heterogeneity was detected in the Ke Shen Qu and Chen Shuo datasets (23, 25), while no significant heterogeneity was observed in the remaining studies.

### 3.7 The effect of TCM on ulcer area

Eight datasets assessed the effect of TCM on ulcer area. Da-Yuan Zhong et al. (18) reported a non-significant reduction using Buyang Huanwu decoction—primarily *Astragalus membranaceus* (Fisch.) Bunge—combined with Western medicine (MD: –1.72; 95% CI: −4.67 to 1.23; I^2^: 96%; n = 184). Three datasets evaluated the rate of ulcer area reduction during TCM intervention. In the first two, *Resina Draconis* (*Daemonorops draco* [Willd.] Blume) applied for 4 and 4.5 weeks significantly increased the probability of achieving ≥70% (RR: 1.29; 95% CI: 1.10–1.59; I^2^: 0%) and ≥50% (RR: 1.38; 95% CI: 1.15–1.66; I^2^: 0%) reductions, respectively (20). In contrast, datasets from Min Chen’s study (17) using Chinese herbal medicine, showed reductions in the probability of minimal to complete ulcer area reduction: <30% (RR: 0.34; 95% CI: 0.21–0.53; I^2^: 76%), 30% (RR: 0.81; 95% CI: 0.71–0.92; I^2^: 0%), and 100% (RR: 0.62; 95% CI: 0.39–0.97; I^2^: 0%). Significant heterogeneity was reported in two datasets (20, 27), while the remaining four showed no significant heterogeneity (17, 18).

### 3.8 The effect of TCM on hospitalization duration

Two studies, each contributing one dataset, assessed the impact of TCM on hospitalization duration. Haoyue Feng et al. (20) used *Resina Draconis* (*Daemonorops draco* [Willd.] Blume) and reported a significant reduction (MD: –9.00; 95% CI: −9.81 to −8.19; I^2^: 0%) with an average intervention duration of 3.75 weeks and a sample size of 213. No significant heterogeneity was observed. Similarly, Xiaoping Wan et al. (22) found a significant effect using *Periploca sepium* Bunge (verified via The World Flora Online) liquid (SMD: –3.68; 95% CI: −5.38 to −1.97; I^2^: 97%) in a larger sample of 408 participants, though significant heterogeneity was present.

### 3.9 The effect of TCM on amputation rate and adverse event

Chen Shuo et al. (25) reported that TCM significantly reduced the odds of amputation by 64% (OR: 0.36; 95% CI: 0.20–0.65), with an average intervention duration of 27 weeks and a sample size of 299. No significant heterogeneity was observed.

Additionally, Da-Yuan Zhong et al. (18) examined the effect of Buyang Huanwu decoction—primarily *Astragalus membranaceus* (Fisch.) Bunge—combined with Western medicine on adverse events. The intervention showed no significant effect (RR: 1.50; 95% CI: 0.57–3.91), and significant heterogeneity was reported.

### 3.10 Statistical approach in included meta-analyses

Across included meta-analyses, fixed-effects models were typically used under low heterogeneity and random-effects otherwise. Examples include: Chen (25) (fixed if *p* > 0.10 and I^2^ < 50%; Egger test + funnel; sensitivity analyses); Tan 2018 (16) (fixed if I^2^ < 50%, random otherwise; funnel plot for primary outcomes); Qu (23) (fixed if I^2^ < 25%, random for 25–85%); Wan (22) (random where *p* < 0.05 and I^2^ > 50%; Begg/Egger/funnel); Zhong (18) (fixed if I^2^ ≤ 50%/*p* > 0.05, random otherwise; funnel plot); and Feng (34) (random when I^2^ > 50% or *p* < 0.10; sensitivity analyses; publication bias not assessed due to <10 studies). These reporting patterns are reflected in Table 1 and our narrative synthesis.

## 4 Discussion

As previously noted, this study aimed to evaluate the effects of various TCM interventions on outcomes related to DFU. The findings indicate that most TCM treatments significantly improve clinical outcomes. Also, based on the form of TCM, topical interventions (e.g., KangFuXin liquid, Resina Draconis, and herbal dressings) showed stronger effects on ulcer area reduction and healing time, whereas oral formulations (e.g., Danggui Sini decoction, Jinhuang powder, Buyang Huanwu decoction) were more frequently associated with improvements in cure and effectiveness rates.

Because all included meta-analyses synthesized RCTs, estimates reflect randomized evidence. However, heterogeneity was common for several outcomes, and publication-bias assessments were variably implemented, which we considered when interpreting consistency and certainty of effects.

Regarding the cure rate, all included datasets showed significant improvement, with the strongest effect observed in the study by Yan-Wu Yang et al. (19) which employed a hydropathic compress using *Astragalus membranaceus*. This treatment contains bioactive compounds such as flavonoids and saponins and is known for its anti-inflammatory and antioxidant properties. Specifically, the hydropathic compress may assist in wound cleansing by removing necrotic tissue, debris, and bacteria, thereby fostering a more favorable healing environment (35). Additionally, Astragalus has immunomodulatory effects, enhancing the immune response and promoting tissue repair (36). Its antioxidant activity helps reduce oxidative stress, a known barrier to wound healing, thus supporting recovery in DFU cases (37). However, while this intervention significantly improved the cure rate, it did not yield a significant effect on the effectiveness rate. This may be due to the significant heterogeneity observed in the effectiveness rate outcome, which was not present for the cure rate, despite similarities in study design. The difference likely reflects variations in outcome measurement methods and assessment timing; distinct outcomes may follow different temporal response patterns (e.g., immediate vs. delayed), resulting in differing levels of observed heterogeneity based on the chosen time points.

The *Compound Cortex Phellodendron amurense* Rupr. (verified via The World Flora Online), examined in Yan-Wu Yang et al. (19), demonstrated a substantial and significant increase in cure rate likelihood. This herbal compound has been reported to possess anti-inflammatory (38) antimicrobial (39) antioxidant, and angiogenic properties, potentially enhancing ulcer healing by reducing inflammation and oxidative stress, inhibiting bacterial growth, stimulating collagen synthesis, and promoting tissue regeneration (38). Additionally, Yan-Wu Yang et al. (19) observed a significant increase in the effectiveness rate following the use of *Phellodendron amurense*, suggesting broader therapeutic potential. Notably, both datasets from Yan-Wu Yang (c and i) had larger sample sizes than other included studies, which enhances the generalizability of their findings.

Furthermore, the dataset from Chen Shuo (25) also showed a large and significant effect size for the cure rate. This dataset incorporated more clinical trials than others and had a notably longer average intervention duration (27 weeks), which may have contributed to the stronger results. Unlike other datasets that used localized treatments, this study included both oral and topical interventions—potentially increasing the overall therapeutic efficacy.

Overall, the findings indicate that various forms of TCM significantly improve cure rates in DFU patients. The magnitude of the effect appears to increase with larger sample sizes and longer intervention durations. Similarly, datasets assessing effectiveness rate also reported significant improvements.

Three datasets—Chen Shuo, Da-Yuan Zhong, and Xiaoping Wan (19, 22, 25), —reported greater effect sizes than others. For Da-Yuan Zhong and Xiaoping Wan, this may be attributed to larger sample sizes and the inclusion of more studies. In Chen Shuo, the extended average intervention duration likely further contributed to the stronger results.

The Da-Yuan Zhong dataset employed Buyang Huanwu decoction, primarily composed of Astragalus membranaceus (Fisch.) Bunge, which, beyond its anti-inflammatory and antioxidant properties, is known to invigorate blood circulation, potentially enhancing oxygen and nutrient delivery to wounds. It also exhibits neuroprotective effects, which may help counter diabetes-induced neuropathy and support nerve repair, improving healing outcomes (40). In the Ke Shen Qu dataset (23) KangFuXin—derived from Periploca sepium Bunge—was used. Its properties include enhancing blood flow, antimicrobial activity, and promotion of angiogenesis and collagen synthesis, all of which support tissue regeneration (41, 42). All included datasets showed a significant reduction in healing time following TCM interventions. Among these, three demonstrated especially strong effect sizes. The Haoyue Feng dataset, rated highly in quality assessment, used Resina Draconis (*Daemonorops* draco [Willd.] Blume), which exhibits anti-inflammatory, antimicrobial, angiogenic, and collagen-stimulating effects, along with wound contraction properties critical for healing (34, 43, 44).

Its larger sample size also contributes to the robustness and generalizability of its findings.

Similarly, the Li Ma dataset (24) also rated as high quality, showed a strong effect in reducing healing time. It used Jinhuang powder (*Curcuma longa* L., verified via The World Flora Online), known for its hemostatic activity—important for controlling bleeding—and its mechanical debriding ability, helping to clear necrotic tissue and bacteria from the wound bed (45). The most pronounced reduction in healing time was observed in the Ke Shen Qu dataset (42), which also used Periploca sepium Bunge. Its potent therapeutic properties, as previously described, likely explain this effect. In contrast, while Xiaoping Wan also employed Periploca sepium Bunge, the observed effect size was smaller. This discrepancy may be due to methodological limitations, such as failure to classify wound severity or adjust for confounding variables like age and gender in the included studies.

Compared with conventional Western treatments such as antibiotics, debridement, or revascularization, TCM interventions appear to act through complementary mechanisms, particularly by enhancing microcirculation, reducing oxidative stress, and modulating immune responses. These actions may explain the additive benefits observed when TCM is used alongside standard care.

Eight datasets have evaluated the effect of TCM on ulcer areas. The result of Da-Yuan Zhong (18) Dataset was not significant, which could be due to the low quality of this study. The following five datasets have measured the risk of ulcer area reduction due to intervention. The two datasets obtained from Haoyue Feng’s article (20) have shown a significant increase in the risk of reducing the rate of ulcer areas. The results showed that using *Resina Draconis; Daemonorops draco (Willd.) Blume* as an intervention causes a more significant increase in the risk of a 50% rate reduction compared to a 70% rate reduction of ulcer areas. A possible reason is the longer intervention duration of the dataset supplement. It also seems evident that the risk of a 50% rate reduction is greater than that of a 70% rate reduction. It should also be mentioned that among the types of TCMs, contraction and reduction of the wound area are prominent features of *Resina Draconis; Daemonorops draco (Willd.) Blume*, which was mentioned in the previous few paragraphs. The following three datasets obtained from the Min Chen study (17) have reported opposite results to the previous datasets and have significantly reduced the risk of decreasing the rate of ulcer areas due to TCM intervention. The results of these datasets seem biased because the studies included are of low quality, including not performing blinding, randomization, and allocation in most of the included studies and not explaining the follow-up process in the studies included in the datasets. The next point is that intervention was done only orally, while in other studies, intervention was done in the form of wound adhesive and topical. It seems that the use of the oral form instead of the topical form has a more significant effect on the improvement in the ulcer area outcome.

### 4.1 Integration with prior meta-analyses

Our umbrella synthesis—spanning 11 meta-analyses—aligns with earlier English-language meta-analyses on DFU adjunctive TCM. For example, meta-analyses evaluating topical formulations (e.g., Kangfuxin liquid, Resina Draconis) (22, 23, 34) report improvements in healing time, healing/“cure” rates, and ulcer area compared with standard care alone, consistent with our findings for topical interventions. Meta-analyses of oral formulations (e.g., Jinhuang powder, Buyang Huanwu decoction, Danggui Sini decoction) (18, 24, 32) similarly report gains in effectiveness and cure rates, again matching the direction of effects we summarize. Because this is an umbrella review, we did not re-pool primary data; rather, we compare and contextualize the reported pooled effects (with CIs and heterogeneity) across meta-analyses, highlighting areas of agreement and inconsistency.

### 4.2 Clinical practice implications

Across the included meta-analyses, TCM was typically evaluated as an adjunct to standard DFU care (e.g., offloading, wound-bed preparation/debridement, infection control, metabolic and vascular optimization). Accordingly, our conclusions pertain to adjunctive use, not replacement of standard care. The clearest signals of benefit appear for topical TCM (healing time, ulcer area), while some oral formulations are more often associated with cure/effectiveness outcomes. Given heterogeneity and variable methodological quality across the contributing meta-analyses, clinicians should apply shared decision-making, consider product quality and local availability, and interpret benefits with appropriate caution. To strengthen international relevance, pragmatic RCTs and meta-analyses from diverse settings outside China—and head-to-head comparisons with other guideline-supported adjuncts—are priorities.

### 4.3 Strength and limitation

To the best of our knowledge, this is the first umbrella meta-analysis to comprehensively examine the effects of various types of TCM on outcomes related to DFU. A wide range of TCM interventions and their effects were systematically analyzed and discussed. However, the study has some limitations. It is also noteworthy that several included meta-analyses were rated as low quality according to AMSTAR2. This raises concerns regarding the robustness of their findings. While we reported their results for completeness, greater weight should be placed on evidence from high- and moderate-quality meta-analyses. Consequently, our conclusions should be interpreted with caution, as the inclusion of low-quality reviews may overestimate treatment effects. Also, limited number of studies addressed key outcomes such as adverse events, amputation rate, and hospitalization duration, restricting the depth of analysis for these endpoints. Additionally, many included studies lacked detailed information on intervention dosage, which hindered more precise evaluation. Future high-quality clinical trials with standardized dosing protocols and extended intervention durations—particularly targeting underrepresented outcomes—are needed to enhance the validity and generalizability of findings. As this was an umbrella review, we synthesized evidence from published meta-analyses without pooled re-analysis; therefore, forest plots were not generated. While consistent with the purpose of umbrella reviews, future studies could strengthen interpretability by re-analyzing primary data and providing graphical synthesis. Another limitation is the absence of protocol registration in PROSPERO or a similar database, which—although this review focused on synthesizing existing meta-analyses—may reduce methodological transparency. Additionally, all included meta-analyses were conducted in China, which introduces a potential geographical bias and may limit generalizability to other healthcare contexts. Furthermore, the majority of the original trials lacked clear reporting of funding sources, creating risk of publication bias or selective reporting. Finally, substantial heterogeneity in TCM formulations, dosages, and intervention durations was observed, which complicates interpretation and comparison across studies.

## 5 Conclusion

Ultimately, most types of TCM appear to positively affect therapeutic outcomes related to DFU, including ulcer area, cure rate, healing time, and effectiveness rate. However, given the variable quality of included evidence, these findings should be interpreted with caution. Also, the use of a topical form of intervention has a more significant impact on the ulcer area outcome than the oral form, but for other outcomes, the oral form seems to have a better performance. Therefore, TCM should be considered and used to treat a DFU patient.

## Data Availability

The original contributions presented in the study are included in the article/Supplementary material, further inquiries can be directed to the corresponding author.
