# Supplementary material for: Traditional Chinese medicine in the management of diabetic foot ulcers: an overview of meta-analyses
Source: Front Med (Lausanne). 2025 Sep 17;12:1651966. doi: 10.3389/fmed.2025.1651966 (PMC12487019; doi:10.3389/fmed.2025.1651966)
Supplement: Supplementary file 1 [file Supplementary_file_1.docx]

**The strategy and terms used to search for related articles are as follows:**

“Traditional Chinese medicine” or “East Asian traditional” or “Drugs Chinese herbal” or “Chinese herbal medicine “or “Tangzu Yuyang Ointment” or “Huang Bai” or “Huang Qi” or “Panax notoginseng” or “Di Huang” or “Dan Shen” or “Wei Di Huang Wan” or “KangFuXin” or astragalus or “buyang huanwu” or yinhuangsan or “Chinese dressing” or “Chinese medicine” or “jinhuang” or “resina draconis” or “Chinese compound medicine” AND “Diabetic foot” or “diabetic feet” or “diabetic foot ulcer” or “foot ulcer” or “Diabetic leg ulcer” or “Diabetic wounds” or “Diabetic skin ulcer” or “foot ulcer” or “skin ulcer” or “leg ulcer” AND ("meta-analysis" OR "meta analysis").

**Limits: no year limits; English language at the umbrella-review level; humans. Reference lists of eligible articles were hand-searched.**
